# Downregulation of Polo-Like Kinase 4 in Hepatocellular Carcinoma Associates with Poor Prognosis

**DOI:** 10.1371/journal.pone.0041293

**Published:** 2012-07-19

**Authors:** Lili Liu, Chris Zhiyi Zhang, Muyan Cai, Jia Fu, George Gong Chen, Jingping Yun

**Affiliations:** 1 State Key Laboratory of Oncology in Southern China, Sun Yat-Sen University Cancer Center, Guangzhou, China; 2 Department of Pathology, Sun Yat-Sen University Cancer Center, Guangzhou, China; 3 Department of Surgery, Prince of Wales Hospital, The Chinese University of Hong Kong, Shatin, N.T., Hong Kong; National Cancer Institute, United States of America

## Abstract

Polo-like kinase 4 (PLK4), belonging to serine/threonine kinase family, is critical for centriole replication and cell cycle progression. PLK4 has been proposed as a tumor suppressor in hepatocellular carcinoma (HCC). However, its expression and significance in HCC have not been well studied. In the present study, we found that PLK4 was markedly downregulated in both HCC cell lines and fresh cancer tissues, using quantitative real-time-PCR and western blot. Immunohistochemistry data also revealed that decreased expression of PLK4 was present in 72.4% (178/246) of HCC tissues, compared with the corresponding adjacent nontumorous tissues. Furthermore, PLK4 expression significantly correlated with clinicopathological parameters, including clinical stage (P = 0.034), serum α-fetoprotein (AFP) (P = 0.019) and tumor size (P = 0.032). Moreover, HCC patients with low PLK4 expression survived shorter than those with high PLK4 expression, as indicated by overall survival (P = 0.002) and disease-free survival (P = 0.012) assessed by the Kaplan–Meier method. In addition, multivariate analysis suggested PLK4 as an independent predictor of overall survival (HR, 0.556; 95%CI, 0.376−0.822; P = 0.003) and disease-free survival (HR, 0.547; 95%CI, 0.382−0.783; P = 0.001). Collectively, our study demonstrated that PLK4 was remarkably downregulated in HCC and could be served as a potential prognostic marker for patients with this deadly disease.

## Introduction

Hepatocellular carcinoma (HCC) is the fifth most prevalent cancer, thirdly leading cancer-related death worldwide [1]. The mortality rate of HCC has been increasing in China since the 1990 s, and HCC has became the second leading cause of cancer death [2]. To date, many risk factors, such as hepatitis B or C viral infection, alcohol consumption, aatoxinB1, and genetic predisposition have been identified as causes of HCC [3], [4], [5], [6]. However, the pathogenic mechanism and inadequacy of early detection of HCC have not been clearly clarified. On the other hand, high incidence of recurrence and metastasis are the primary reasons of poor prognosis of HCC [7]. As a result, a large series of investigations are focused on the discovery of biological markers useful for HCC diagnosis and prognostic prediction to provide scientific guidance to clinical management.

Polo-like kinases (PLKs) play essential roles in cell cycle progression [8], [9], [10], [11], [12], and DNA damage response [13], [14]. Polo-like kinase 4 (PLK4), originally identified in *Drosophila* as a serine/threonine kinase and mapped to chromosome 4 q28 which is a region frequently associated with loss of heterozygosity (LOH) in hepatoma [15], is essential for centriole duplication and cell cycle progression [16], [17]. PLK4 gradually increases from G1 phase and peaks in mitosis, suggesting that its expression is regulated in a cell cycle dependent fashion [18], [19]. In human cancers, PLK4 is differently expressed. For example, PLK4 was demonstrated to be downregulated in HCC [20], [21], but upregulated in colorectal cancer [22]. Dysregulation of PLK4 caused disturbance of centrosome duplication, which may ultimately resulted in occurrence of tumor [15]. Findings that PLK4 was reduced during hepatocarcinogenesis due to the promoter hypermethylation [23], and that decrease of PLK4 contributed to HCC development in PLK4 mutant mice [24] suggest reduced PLK4 expression may associate with HCC carcinogenesis. Furthermore, PLK4 homozygous null mice were embryonic lethal at E7.5 with a marked increase in mitotic and apoptotic cells [19]. Less chances of bearing spontaneous lung and liver cancer were recorded in PLK4^+/+^ mice, compared with PLK4^+/−^ littermates [20].

In this study, we examined PLK4 expression in HCC cell lines and human tissues, analyzed the correlation between PLK4 expression and clinicopathological characteristics, and determined the role of PLK4 in HCC prognostic prediction. Our data indicated that PLK4 was remarkably decreased in HCC and could be served as a promising biomarker of prognosis.

## Materials and Methods

### Cell Culture

L02, MiHA and Huh7 cell lines were purchased from American Type Culture Collection (ATCC, Manassas, VA). SMMC-7721, Bel-7404, Bel-7402 and QSG-7703 cell lines were obtained from the Type Culture Collection Cell Bank, Chinese Academy of Science Committee (Shanghai, China). L02 was maintained in RPMI 1640 with 15% of fetal bovine serum (FBS), 100 U/ml of penicillin, and 100 U/ml of streptomycin. SMMC-7721, Bel-7404, Bel-7402 and QSG-7703 cells were cultured in RPMI 1640 with 10% of fetal bovine serum (FBS), 100 U/ml of penicillin, and 100 U/ml of streptomycin. MiHA and Huh7 cells were cultured in Dulbecco modified Eagle medium (DMEM) containing 10% of fetal bovine serum (FBS), 100 U/ml of penicillin, and 100 of U/ml streptomycin. All cell lines were incubated in a humidified atmosphere of 5% CO_2_ and 95% air at 37°C.

**Figure 1:**
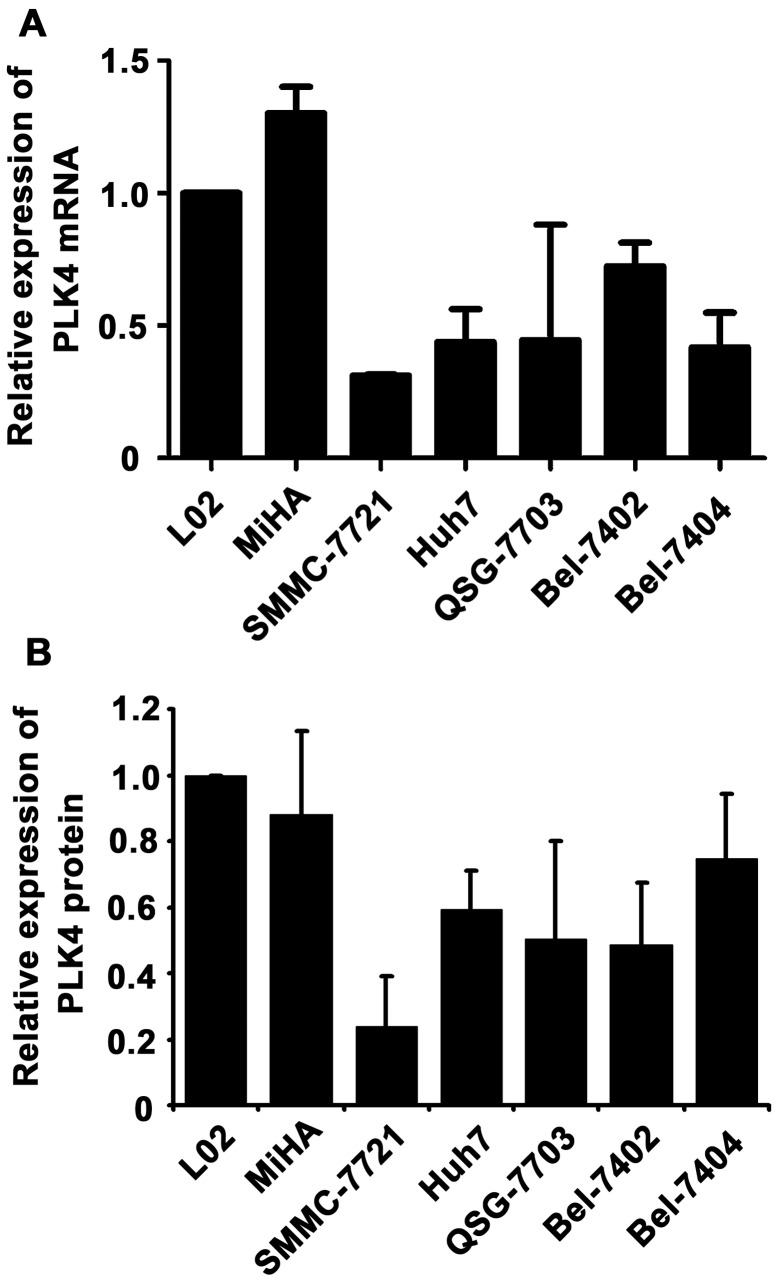
The mRNA and protein expression of PLK4 in HCC cell lines by qRT-PCR and western blot. PLK4 mRNA (**A**) and protein (**B**) expression was examined in immobilized liver cell lines and HCC cells by qRT-PCR and western blot. The liver cell line L02 served as control. The PLK4 levels were normalized to β-actin.

#### Patients and tissue specimens

All HCC specimens along with complete clinical and pathological data were obtained from 246 HCC patients who underwent surgical resection at Sun Yat-Sen University Cancer Center (SYSUCC), Guangzhou, China, between Feb 1997 and Dec 2001. Twenty paired HCC and corresponding adjacent nontumorous tissues immersed in RNAlater (Ambion, Inc., USA) immediately after surgical resection and stored at −80°C were subjected to quantitative real-time RT-PCR and western blot. 219 males (91.3%) and 27 females (8.7%) comprise of the 246 patients aged from 14 to 78 years (median age is 48). None of the patients had received adjuvant therapies before surgery. Tumor stage was defined according to tumor-node-metastasis (TNM) classification of the American Joint Committee on International Union against Cancer. Tumor differentiation was assessed according to Edmonson and Steiner grading system. The use of tissues for this study has been approved by the Institute Research Medical Ethics Committee of SYSUCC.

**Figure 2:**
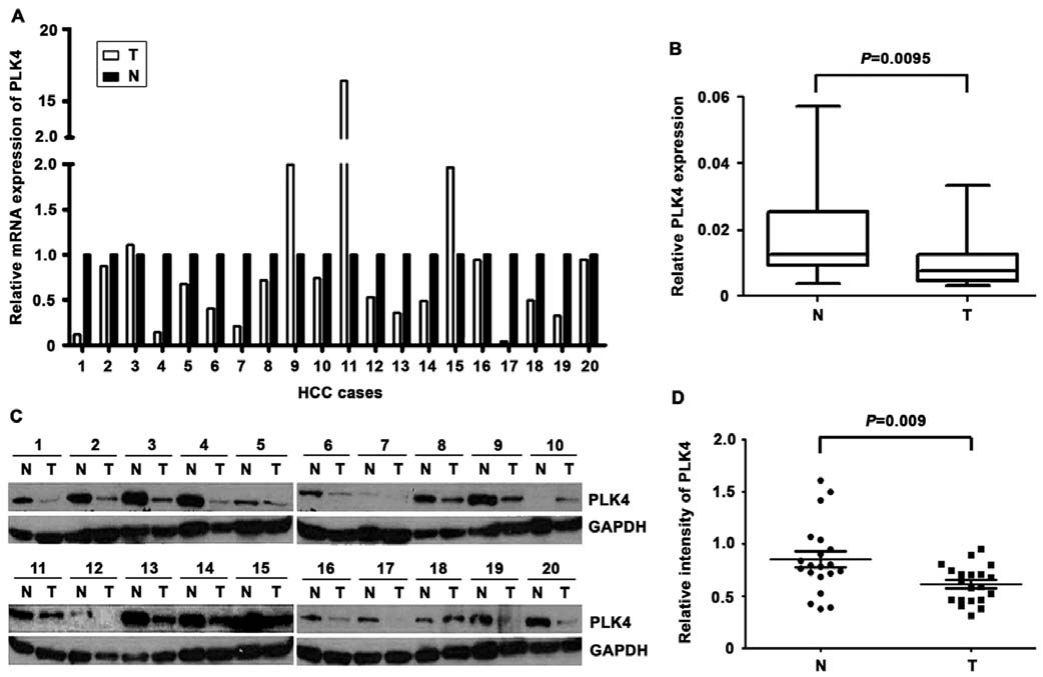
The mRNA and protein expression of PLK4 in HCC by qRT-PCR and western blot. A. Down-regulated expression of PLK4 mRNA was detected by qRT-PCR in 13 out of 20 HCC cases. The relative PLK4 mRNA expression was indicated by histogram (T, tumor tissue; N, the corresponding adjacent non-tumor liver tissues). **B.** The PLK4 mRNA levels was significantly decreased in HCC as determined by the Wilcoxon matched paired test. **C.** Decreased expression of PLK4 protein was shown by western blot in 15 out of 20 HCC tumor tissues. The relative PLK4 protein expression was shown. GAPDH was used as loading control. **D.** Intensity of PLK4 normalized to GAPDH was indicated Wilcoxon matched paired test.

**Figure 3:**
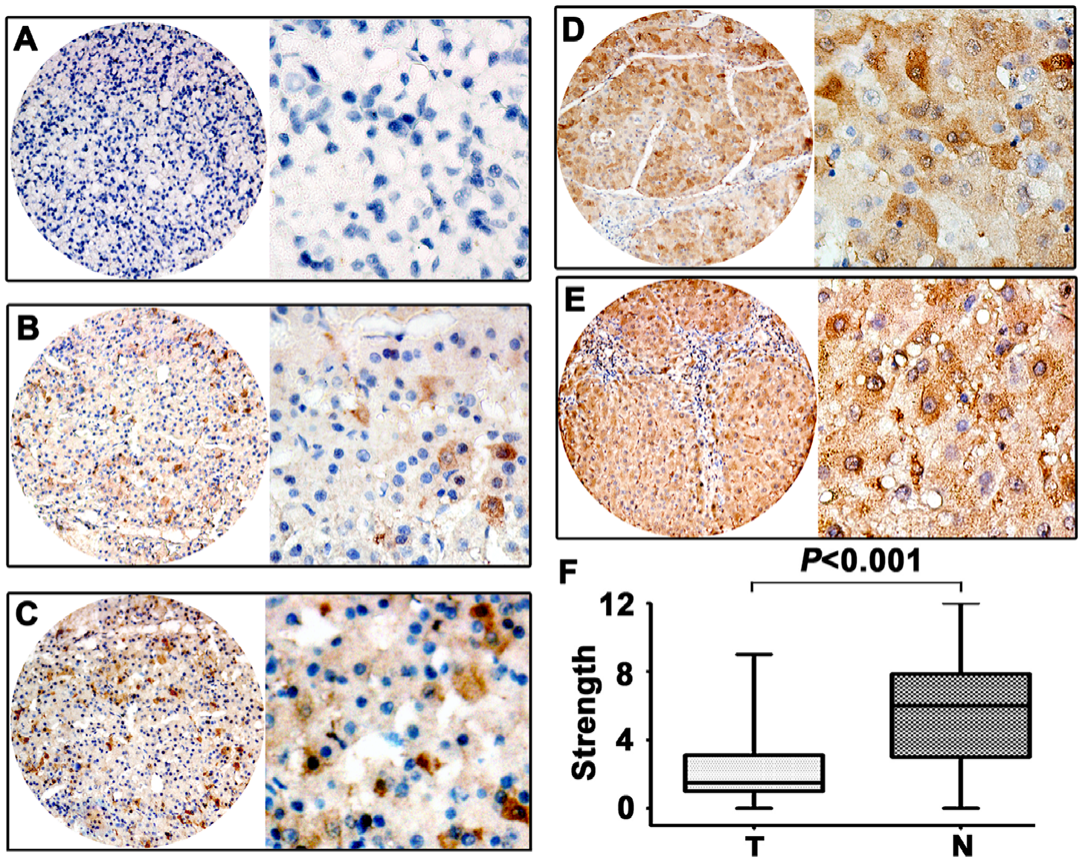
The expression of PLK4 in HCC by IHC. The micrographs showed negative (**A**), weak (**B**), moderate (**C**), strong (**D**) staining of PLK4 in tumor tissues, as well as strong (**E**) staining of normal liver tissues. (Left panel: magnification ×40; Right panel: magnification ×400.) **F.** Reproducibility of the measurement in all 246 patients was calculated by the Wilcoxon matched paired test.

#### Tissue microarray (TMA) construction

According to the method described previously [25], we constructed the tissue microarray, containing of 246 HCC and adjacent nontumorous tissues. In brief, all specimens were fixed in 10% formalin and embedded in paraffin. The corresponding histological HE-stained sections were reviewed by two pathologists to mark out representative areas. Using a tissue arraying instrument (Beecher Instruments, Sliver Spring, MD), each tissue core with a diameter of 0.6 mm was punched from the marked areas and re-embedded.

**Table 1:** Correlation between PLK4 expression and clinicopathologic parameters in HCC.

Variable	PLK4 protein				
	All cases	Low expression	High expression	x^2^	*P* value^a^
Age(years)^b^				0.021	0.886
<48	120	63 (52.5%)	57 (47.5%)		
≥48	126	65 (51.6%)	61 (48.4%)		
Gender				0.151	0.698
Female	27	15(55.6%)	12(44.4%)		
Male	219	113(51.6%)	106(48.4%)		
HBsAg				0.729	0.393
Yes	212	108(50.9%)	104 (49.1%)		
No	34	20(58.8%)	14 (41.2%)		
AFP (ng/ml)				5.544	**0.019**
<20	104	45(43.3%)	59 (56.7%)		
≥20	142	83 (58.5%)	59 (41.5%)		
Liver cirrhosis				0.066	0.798
Yes	177	93 (52.5%)	84(47.5%)		
No	69	35 (50.7%)	34 (49.3%)		
Tumor size (cm)				4.603	**0.032**
<5	118	53 (44.9%)	65 (55.1%)		
≥5	128	75 (58.6%)	53(41.4%)		
Tumor multiplicity				0.363	0.547
Single	130	70 (53.8%)	60(46.2%)		
Multiple	116	58 (50.0%)	58 (50.0%)		
Differentiation				5.216	0.157
Well (I)	32	21(65.6%)	11 (34.4%)		
Moderate (II)	104	48 (46.2%)	56 (53.8%)		
Poor (III)	95	53 (55.8%)	42 (44.2%)		
Undifferentiation (IV)	15	6 (40.0%)	9(60.0%)		
Stage				8.665	**0.034**
I	22	6 (27.3%)	16 (72.7%)		
II	109	55 (50.5%)	54 (49.5%)		
III	87	48 (55.2%)	39 (44.8%)		
IV	28	19 (67.9%)	9 (32.1%)		
Vascular invasion				0.661	0.416
Yes	140	76(54.3%)	64(45.7%)		
No	106	52(49.1%)	54 (50.9%)		

a Chi-square test;

b patients were divided according to the median age; AFP, alpha-fetoprotein; HBsAg, hepatitis B surface antigen; PLK4, polo-like kinase 4.

#### RNA preparation and quantitative real-time PCR

Total RNA was extracted from 20 pairs of HCC samples, following the Trizol reagent (BIOO Scientific Co., USA) manufacturer’s instruction. mRNA was reversed to cDNA by M-MLV Reverse Transcriptase (Promega Inc., USA). The levels of PLK4 and β-actin were measured by SYBR green-based real-time PCR using the Stratagene Mx3000P Real-Time PCR system. Primers were designed as follows: PLK4, Forward: AATCAAGCACTCTCCAATC and Reverse: TGTGTCCTTCTGCAAATC; β-actin, Forward: TGGCACCCAGCACAATGAA and Reverse: CTAAGTCATAGTCC GCCTAGAAGCA. Conditions were set as follows: one cycle of 95°C for 10 min, followed by 40 amplification cycles at 95°C for 10 s, annealing at 60°C for 20 s and elongation at 72°C for 15 s. Using the comparative threshold cycle (2^−ΔΔCt^) method [26], the relative expression of PLK4 in HCC were normalized to the endogenous β-actin.

**Figure 4:**
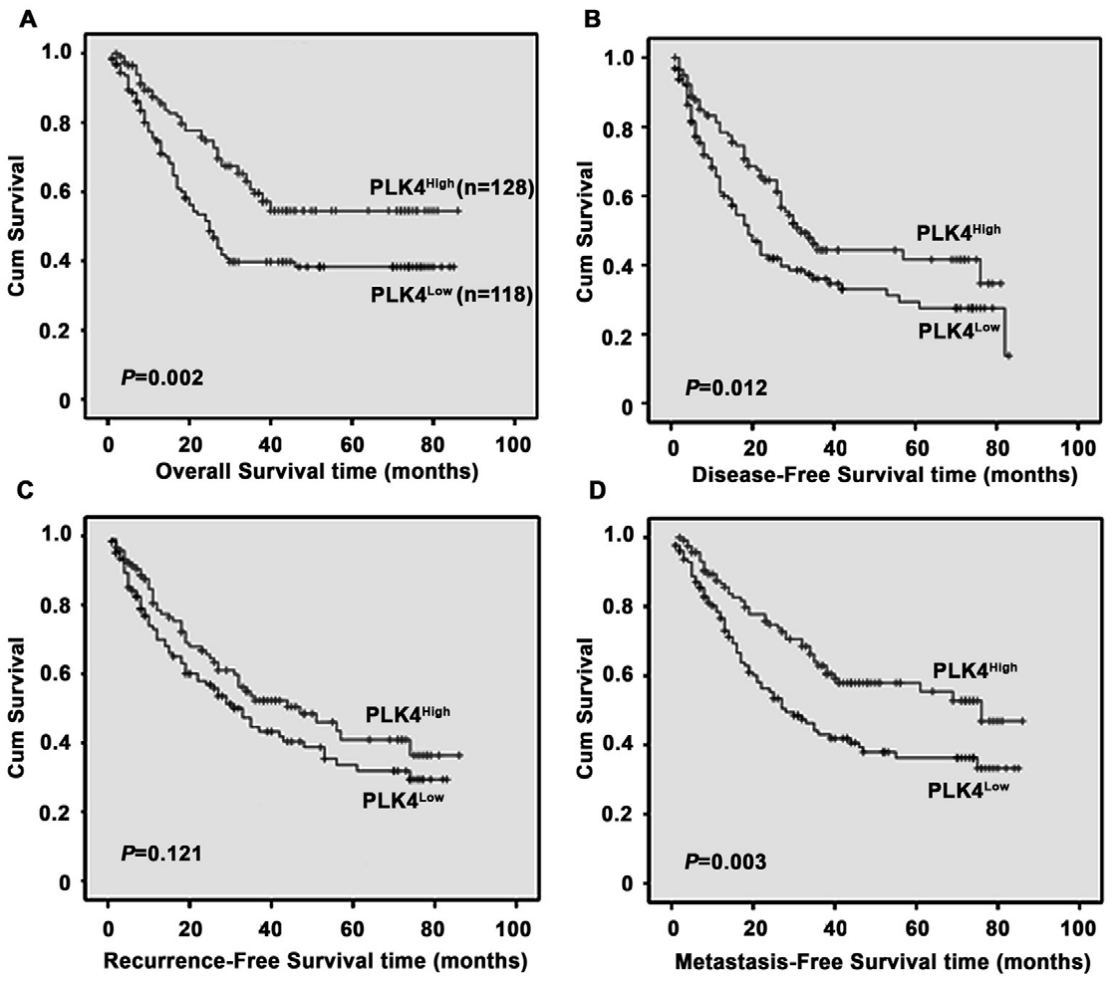
Relationship between PLK4 expression and HCC prognosis. PLK4 protein level showed prognostic role in overall survival (**A**), disease-free survival (**B**) and metastasis-free survival (**D**), but not for recurrence-free survival (**C**), as indicated by Kaplan-Meier analysis.

#### Western blot

Total protein was extracted from 20 pairs of HCC fresh tissues. 30 ug of protein was loaded onto 8% SDS-PAGE and transferred to PVDF membranes. After blocking, the membranes were incubated with primary antibody against PLK4 (1:2000 dilutions, rabbit anti-PLK4, Epitomics Biotechnology, Inc., USA). The membranes were then incubated with horseradish peroxidase-linked mouse anti-rabbit antibody (at a 1:3000 dilution, Santa Cruz Biotechnology, Inc., Santa Cruz, Calif., USA). GAPDH was served as a loading control.

**Figure 5:**
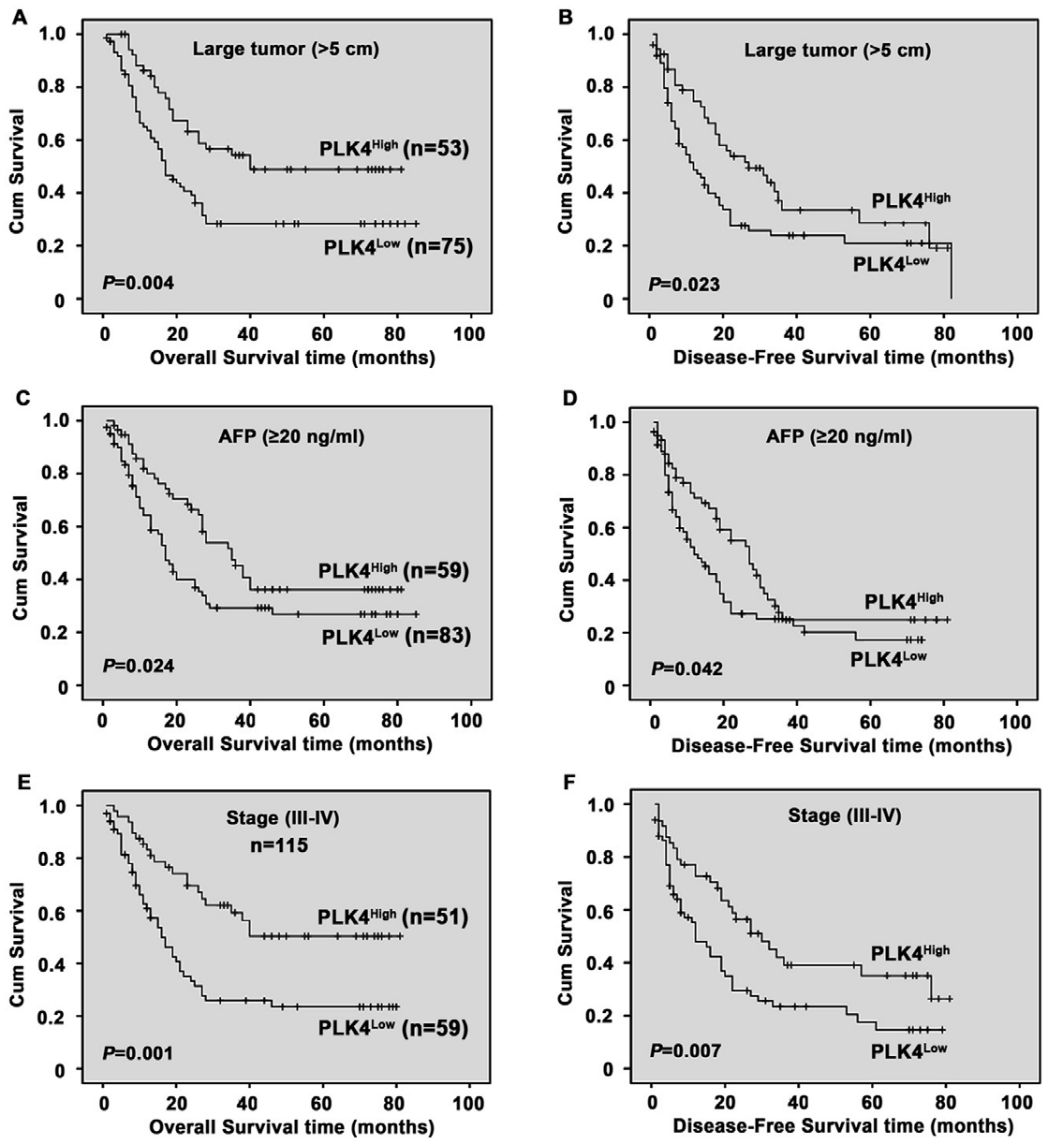
Analysis of PLK4 protein expression in relation to overall survival and disease-free survival of subclassified HCC patients. In term of overall survival and disease-free survival, subgroup analysis indicated that PLK4 had prognostic role when classified by the following variables: tumor size (A–B), AFP (C–D), stage (III–IV) (E–F) (log-rank test).

#### Immunohistochemistry

Immunohistochemistry (IHC) analysis for PLK4 was performed using a standard two-step method [27]. TMA sections were baked overnight at 37°C, and then deparaffinized and rehydrated. Slides were boiled in Ethylene Diamine Tetraacetic Acid (EDTA; 1 mmol/L; PH 8.0) in a pressure cooker for antigen retrieval. Subsequently, slides were incubated overnight at 4°C with PLK4 antibody (1:1600 dilution, goat polyclonal antibody, Santa Cruz Biotechnology, Inc., Santa Cruz, Calif., USA). After rinsed with PBS, the slides were incubated with a secondary antibody and stained with 3, 3-diaminobenzidine tetrahydrochloride (DAB). Finally, the slides were counterstained with Mayer’s hematoxylin. Slides immunoreacted with PBS were used as the negative controls.

**Table 2:** Univariate analysis of different prognostic factors in 246 HCC patients.

Variable	Overall survival		Disease-free survival	
	HR (95% CI)	*P* value	HR (95% CI)	*P* value
Age (years)	1.160(0.801−1.679)	0.432	1.043(0.741−1.469)	0.807
Gender	1.262(0.677−2.352)	0.464	0.856(0.507−1.446)	0.561
HBsAg	0.902(0.524−1.554)	0.711	1.245(0.727−2.132)	0.424
AFP (ng/ml)	2.951(1.933−4.506)	<**0.001**	2.725(1.865−3.982)	<**0.001**
Liver cirrhosis	1.110(0.731−1.685)	0.626	1.230(0.829−1.824)	0.304
Tumorsize (cm)	2.065(1.400−3.046)	<**0.001**	2.142(1.497−3.064)	<**0.001**
Tumormultiplicity	2.830(1.908−4.198)	<**0.001**	2.595(1.809−3.723)	<**0.001**
Differentiation	1.918(1.493−2.465)	<**0.001**	1.854(1.473−2.333)	<**0.001**
Stage	1.637(1.302−2.058)	<**0.001**	1.612(1.308−1.987)	<**0.001**
Vascularinvasion	5.705(3.401−9.569)	<**0.001**	3.991(2.597−6.134)	<**0.001**
PLK4	0.553(0.379−0.806)	**0.002**	0.648(0.458−0.916)	**0.014**

CI, confidence interval; HR, hazard ratio; AFP, alpha-fetoprotein; HBsAg, hepatitis B surface antigen; PLK4,polo-like kinase4.

**Table 3:** Multivariate analysis of overall and disease-free survival rates of HCC patients.

Variable	β	SE	Hazard ratio (95%CI)	*P* value
**Overall survival**				
Tumor size	0.220	0.211	1.246 (0.824−1.884)	0.297
Stage	0.011	0.157	1.012 (0.744−1.375)	0.942
Tumor multiplicity	0.294	0.259	1.342 (0.808−2.229)	0.256
Vascular invasion	1.275	0.315	3.578 (1.929−6.638)	**<0.001**
AFP	0.841	0.221	2.318 (1.504−3.573)	**<0.001**
Differentiation	0.264	0.130	1.302 (1.009−1.680)	**0.042**
PLK4	−0.587	0.200	0.556(0.376−0.822)	**0.003**
**Disease-free survival**				
Tumor size	0.345	0.192	1.412(0.969−2.058)	0.073
Stage	0.22	0.146	1.022 (0.767−1.362)	0.881
Tumor multiplicity	0.347	0.239	1.415(0.885−2.261)	0.147
Relapse	1.051	0.191	2.860(1.966−4.162)	**<0.001**
Vascular invasion	0.869	0.279	2.384 (1.380−4.119)	**0.002**
AFP	0.773	0.199	2.167 (1.468−3.198)	**<0.001**
Differentiation	0.169	0.128	0.184 (0.921−1.522)	0.189
PLK4	−0.603	0.183	0.547 (0.382−0.783)	**0.001**

β, Regression coefficient; SE, standard error; CI, confidence interval; AFP, alpha-fetoprotein; PLK4, polo-like kinase4.

#### Immunohistochemistry evaluation

Semi-quantitative IHC detection was used to determine the PLK4 protein levels. Using the H-score method [28], we multiplied the percentage score by the staining intensity score. The percentage of positively-stained cells was scored as “0” (0%), “1” (1%–25%), “2” (26%–50%), “3” (51%–75%), “4” (76%–100%). Intensity was scored as “0” (negative staining), “1” (weak staining), “2” (moderate staining), and “3” (strong staining).The median H-score was chosen as cutoff point to separate “high PLK4 expression” (H-score>median) from “low PLK4 expression” (H-score ≤ median) tumor samples.

#### Statistical analysis

Statistical analyses were performed using the SPSS 16.0 software (SPSS, Chicago, IL, USA). The Students’ *t* test was used for comparison between groups. The χ^2^ test was performed to analyze the correlation between PLK4 expression and clinicopathological parameters. The Kaplan-Meier method (the log-rank test) was used for survival curves. Cox regression model with stepwise manner (forward, likelihood ratio) was utilized to perform a multivariate analysis. *P*<0.05 (two-tailed) was considered statistically significant.

## Results

### The Expression of PLK4 in HCC Cell Lines

To examine the expression of PLK4 in HCC, we firstly detected its mRNA level in immortalized liver cell lines and HCC cell lines. According to the results of qRT-PCR, levels of PLK4 mRNA in HCC cell lines were noticeably lower than those in immortalized liver cells (Fig. 1A). We next determined the protein expression of PLK4. As depicted in the result of western blot, PLK4 was markedly downregulated in most of the tested HCC cells, compared to that of L02 cells (Fig. 1B).

#### The mRNA and protein levels of PLK4 in HCC tissues

We next examined PLK4 expression in 20 paired HCC and the corresponding adjacent nontumor tissues, using qRT-PCR and western blot. In 13 out of 20 cases, PLK4 mRNA was downregulated in tumor tissues, compared with the adjacent nontumorous tissues (Fig. 2A). PLK4 mRNA expression was remarkably higher in 3 cases while remained unchanged in the rest samples. Consistently, in 75% of cases, the protein levels of PLK4 in HCC tissues were dramatically lower than those in the nontumorous tissues (Fig. 2C), as depicted in the result of western blot, using the same HCC samples for qRT-PCR. The altered expression of PLK4 between tumor and adjacent nontumor tissues appeared statistically significant (Fig. 2B&D).

#### The relationship between PLK4 expression and clinicopathological parameters

IHC was performed to assess the expression of PLK4 in 246 paraffin-embedded HCC tissues. Results revealed that PLK4 expression was mainly present in the cytoplasm of cancer cells (Fig. 3A–E) Scattered staining of PLK4 in nuclear was also observed. As indicated by Figure 3F, low PLK4 expression in tumor tissue was found in 175 out of 246 (71.1%) cases.

The relationship between PLK4 expression and clinicopathological parameters was further analyzed. Significant correlations were found between PLK4 expression and three parameters including clinical stage (*P* = 0.034), serum AFP positive (*P* = 0.019) and tumor size (*P* = 0.032). HCC patients with low PLK4 expression had a higher tendency to be with advanced stage, high level of serum AFP and large-size tumor. There were no statistical connections between PLK4 expression and the rest clinicopathological parameters, such as age, gender, HBsAg, cirrhosis, differentiation and vascular invasion (*P*>0.05, Table 1).

#### The association of low PLK4 expression in HCC with poor survival

The association between PLK4 expression in HCC and the survival of selected patients was analyzed with Kaplan-Meier survival analysis. Patients with low PLK4 expression were likely to be with significantly shorter overall survival (*P* = 0.002, Fig. 4A), disease-free survival (*P* = 0.012, Fig. 4B) and metastasis-free survival (*P* = 0.003, Fig. 4C), but not recurrence-free survival (*P* = 0.121, Fig. 4D).

Since PLK4 expression was significantly corrected to AFP, tumor size and clinical stage, we further determined the relationship between PLK4 expression and the survival of patients subclassified as ‘large tumor’, ‘AFP (≥20 ng/ml)’ and ‘Stage (III–VI)’. As showed by Figure 5, patients in subclassified groups with low PLK4 expression survived shorter than those with high PLK4 expression.

#### Univariate and multivariate analyses of prognostic variables in HCC Patients

We next evaluated the expression of PLK4 and other clinicopathologic parameters on prognosis of HCC, using univariate analyses. Results indicated that PLK4, as well as serum AFP level, tumor size, tumor multiplicity, tumor differentiation, clinical stage and vascular invasion, was responsible for efficacy of surgical treatment in HCC patient, by showing that PLK4 expression was significantly associated with overall survival (*P* = 0.002) and disease-free survival (*P* = 0.014) of HCC patients (Table 2).

Furthermore, PLK4 expression and those clinicopathologic variables significant in univariate analysis (i.e., serum AFP level, tumor size, tumor multiplicity, tumor differentiation, clinical stage and vascular invasion) were further evaluated in multivariate analysis. Results suggested that PLK4 was also an independent predictor for overall survival (HR: 0.556, 95% CI: 0.376−0.822, *P* = 0.003) and disease-free survival (HR: 0.547, 95% CI: 0.382−0.783, *P* = 0.001) of HCC patients (Table 3).

## Discussion

Polo-like kinases (PLKs) are important regulators of cell cycle progression, mitosis, cytokinesis, and DNA damage response [8], [9], [10], [11], [12], [14]. A previous study showed that both mRNA and protein expressions of PLK4 gradually decreased from normal liver to HCC [24]. In this study, to elucidate the clinical role of PLK4 in HCC, we applied TMA and IHC to examine its expression in a cohort of Chinese patients. A significant decline of PLK4 expression was observed in HCC tissues, compared with the adjacent nontumorous tissues. To our knowledge, this is the first study to analyze PLK4 expression in HCC using TMA-based IHC method.

Clinically, correlation of PLK4 expression with clinicopathological parameters was also determined in previous studies. Reduced expression of PLK4 was reported to connect with age in colorectal tumor [22]. Decreased expression of PLK4 in HCC was associated with larger tumor size, indicating that the loss of PLK4 may facilitate HCC growth. This adds to the previous findings reporting the central role of PLK4 in mitotic regulation [8] and centriole duplication [16]. In the present study, we found low expression of PLK4 in HCC was significantly associated with other malignant tumor characteristics, such as advanced stage and high serum AFP, indicating that PLK4 might be served as a hallmark of advanced stage tumors. This could be supported by the findings that constitutive expression of murine PLK4 suppressed cell growth [18], and that downregulation of PLK4 antagonized the antiproliferative and proapoptotic effects in nontumor cells [21]. Collectively, PLK4 may have antitumor properties. Monitoring the expression dynamics of PLK4 may contribute to characterize patients as frequently monitored ones or adjuvant therapy given ones.

In results of Kaplan-Meier survival analysis, we found that patients with high PLK4 expression had longer survival. This might be explained by the findings that high PLK4 expression positively correlated with a higher proliferation index [29], and that high proliferative tumors could better response to therapy. Furthermore, Cox regression analysis suggested PLK4 as an independent prognostic factor, indicating that PLK4 may be a pivotal modulator involved in cancer development. This could be supported by other reports showing that decreased PLK4 led to dysregulation of cell proliferation [29], and that PLK4^−/−^ embryos were arrested at E7.5 and ultimately dead [19]. On the other hand, recent studies provided a comprehensive understanding of PLK4’s role in tumor progression and cell differentiation. Elderly PLK4^+/−^ mice usually had more chances to develop spontaneous liver and lung cancer [20]. Furthermore, PLK4 controlled the differentiation of trophoblast stem cells into trophoblast giant cells *in vitro* [30]. Collectively, downregulation of PLK4 in tumor tissues may facilitate tumor progression, subsequently leading to poor prognosis.

Stratified survival analysis of HCC, according to clinical stage, tumor size and serum AFP, evaluated PLK4 expression to be closely correlated with survival of HCC patients with stage III–IV, larger tumor size and high-level AFP. This suggested that downregulation of PLK4 in HCC could be of clinical use for distinguishing a set of patients with poor prognosis. Tumor differentiation and vascular invasion and serum AFP are the most commonly agreed survival indices for affecting the prognosis of HCC patients [31], [32], [33]. But these three parameters have different types of limits in offering critical information affecting patient prognosis. In this study, our results indicated PLK4 expression evaluated by IHC could be used as an additional tool in identifying those patients at risk of HCC progression.

Taken together, our study provides compelling clinical evidence that PLK4 can be served as an independent prognostic marker for overall survival and disease-free survival in HCC. Decreased PLK4 expression in HCC is significantly related to tumor size, serum AFP, and clinical stage. Although the current results which are based on a cohort of Chinese patients should be further confirmed in other HCC cohorts, our findings suggest PLK4 as a new and promising prognostic biomarker for HCC progression and prognosis.
